# Towards Elucidating the Rotary Mechanism of the Archaellum Machinery

**DOI:** 10.3389/fmicb.2022.848597

**Published:** 2022-03-21

**Authors:** João Nuno de Sousa Machado, Sonja-Verena Albers, Bertram Daum

**Affiliations:** ^1^Molecular Biology of Archaea, Faculty of Biology, Institute of Biology II, University of Freiburg, Freiburg, Germany; ^2^Spemann Graduate School of Biology and Medicine, University of Freiburg, Freiburg, Germany; ^3^Living Systems Institute, University of Exeter, Exeter, United Kingdom; ^4^College of Life and Environmental Sciences, University of Exeter, Exeter, United Kingdom

**Keywords:** archaea, archaellum motor, cell motility, type IV pili, archaella, molecular motors

## Abstract

Motile archaea swim by means of a molecular machine called the archaellum. This structure consists of a filament attached to a membrane-embedded motor. The archaellum is found exclusively in members of the archaeal domain, but the core of its motor shares homology with the motor of type IV pili (T4P). Here, we provide an overview of the different components of the archaellum machinery and hypothetical models to explain how rotary motion of the filament is powered by the archaellum motor.

## Introduction

Rotary nanomachines play an essential role in virtually all living cells. A particularly famous example is the ubiquitous ATP synthase, which converts a proton gradient into ATP by rotary catalysis (Itoh et al., 2004). Other gyrating molecular machines, such as flagella, gliding motors, and archaella, drive cellular propulsion (Berg, 2003; Albers and Jarrell, 2015; Shrivastava et al., 2015).

Among these propulsive molecular machines, the archaellum is particularly interesting, considering its evolutionary history. The archaellum is part of a superfamily of molecular machines called type IV filaments (TFF), which include pili [e.g., type IV pili (T4P)] and secretion systems [e.g., type II secretion (T2SS); Berry and Pelicic, 2015]. While some T4P drive cellular motility through cycles of extension and retraction (Mattick, 2002), the archaellum is the only known member of the TFF superfamily with a rotating filament.

In this review, we summarise the current knowledge about the biophysics and structure of the archaellum machinery and present a hypothetical model describing its mechanism.

## The *arl* Operon

**Figure 1:**
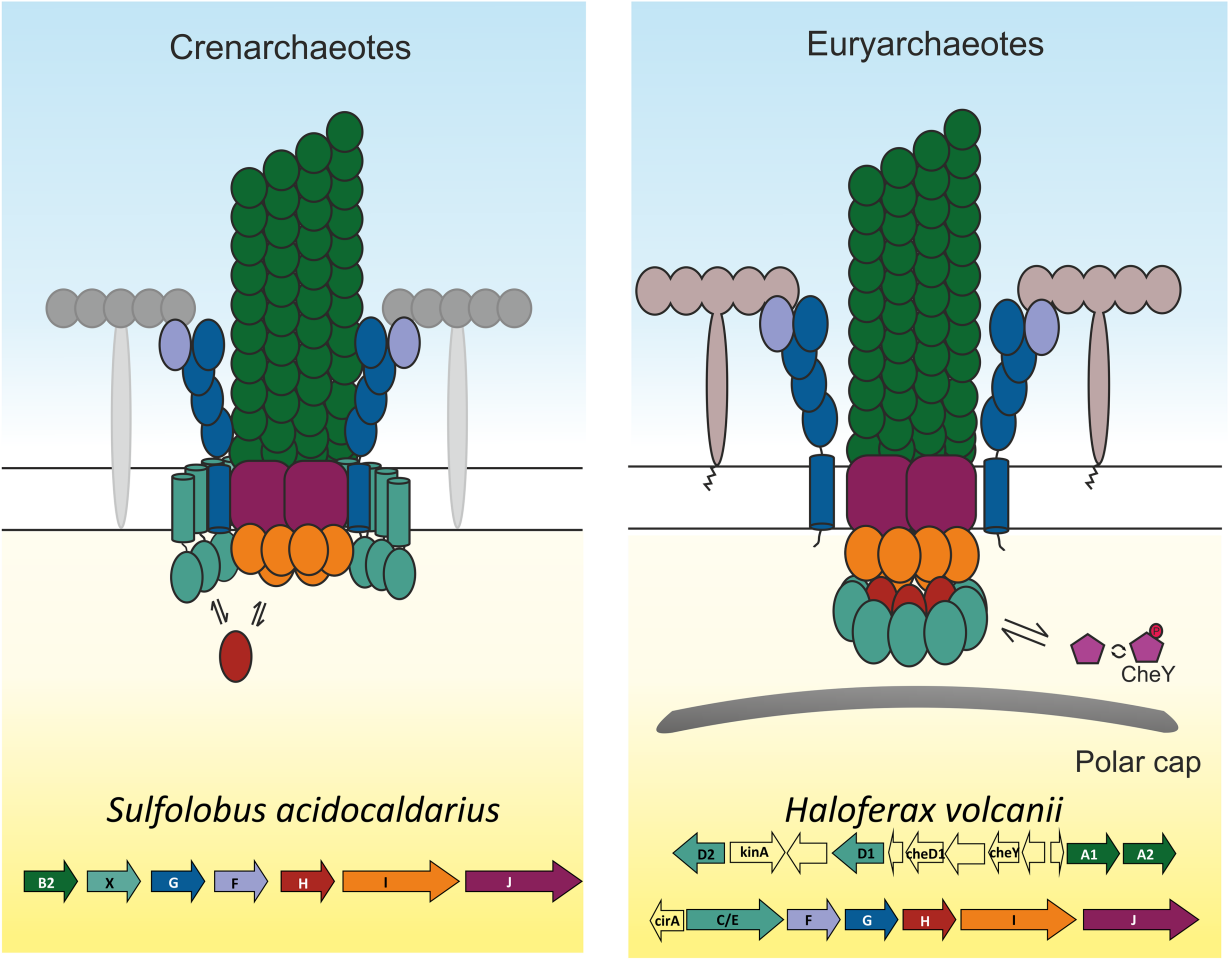
The archaellum machinery in Cren- and Euryarchaeota. The structural proteins of the archaellum are encoded by genes organised in the *arl* cluster. The *arl* cluster differs between Crenarchaeota and Euryarchaeota in three main aspects: Crenarchaeota usually encode a single archaellin, with multiple present in the euryarchaeal arl cluster; the order of the genes *arlF* and *arlG* is different between the two phyla. The gene *arlX* is present only in Crenarchaeota and is replaced with *arlCDE* in Euryarchaeota (Desmond et al., 2007). ArlCDE in Euryarchaeota is thought to be linked to the chemotaxis machinery. ArlCDE is absent in Crenarchaeota, which also lack chemotaxis. The pre-archaellin peptidase PibD/ArlK is also essential for archaella biogenesis, but this gene is frequently found elsewhere in the genome. Upon expression of the cluster, the archaellum motor complex self assembles at the membrane.

Archaella are found across various archaeal phyla, from the relatively well-characterised Euryarchaeota and Crenarchaeota to less understood organisms, such as the putative ectosymbionts of the DPANN superphylum (Jarrell et al., 2021). The biogenesis of a functional archaellum requires the expression of 7–15 genes, which are usually organised in a cluster—the *arl* operon—plus a membrane-embedded aspartic acid protease, often encoded elsewhere in the chromosome (Desmond et al., 2007; Pohlschröder et al., 2018; Jarrell et al., 2021). The aspartic acid protease ArlK/PibD is essential for motility, as it is responsible for cleavage of the class III signal peptide from the filament-forming archaellin subunits (Bardy and Jarrell, 2002, 2003; Albers et al., 2003). The archaellins are usually encoded by gene(s) at the start of the *arl* cluster, with the remaining genes in the cluster coding for proteins of the archaellum motor complex (Figure 1). The motor complex consists of the ATPase ArlI, which powers the assembly and rotation of archaella; a putative switch protein ArlH (Reindl et al., 2013; Chaudhury et al., 2018); ArlJ, a polytopic membrane-embedded platform protein (Ghosh and Albers, 2011); and the pseudoperiplasmic stator proteins ArlF and ArlG (Banerjee et al., 2015; Tsai et al., 2020; Umrekar et al., 2021). In Crenarchaeota, a predicted membrane protein called ArlX is thought to form a cytosolic ring that serves as a scaffold for the motor (Banerjee et al., 2012). In Euryarchaeota, ArlX is likely replaced by ArlCDE and in Thaumarchaeota by a yet to be identified protein (Desmond et al., 2007; Jarrell et al., 2021).

Apart from the proteins directly involved in archaellum biogenesis, a subset of archaea belonging to Euryarchaeota or Thaumarchaeota is equipped with a chemotaxis sensory system (Briegel et al., 2015). The absence of chemotaxis in Crenarchaeota has been suggested to be correlated with the lack of *arlC/D/E* genes in this phylum (Albers and Jarrell, 2018). The chemotaxis genes in archaea resemble those found in Bacteria and, in fact, their origin appears to be the result of horizontal gene transfer (HGT). An archaeal-specific protein CheF provides the link between the bacteria-like chemotaxis machinery and the archaellum motor complex (Schlesner et al., 2009; Briegel et al., 2015).

## The Archaellum Filament

The archaellum machinery consists of a helical filament driven by a motor complex. Even before the formal definition of the domain Archaea, researchers described the filament as a rotating structure that allowed for both forward and reverse movement in *Halobacterium salinarum* (Alam and Oesterhelt, 1984; Woese et al., 1990; Marwan et al., 1991). This filament can rotate either clock- or counter-clockwise, maintaining a chiral super-helical structure regardless of the direction of rotation (Marwan et al., 1991; Kinosita et al., 2016). Clockwise rotation of the archaellum enables the cell to swim forward, while anticlockwise rotation propels the cell backwards.

**Figure 2:**
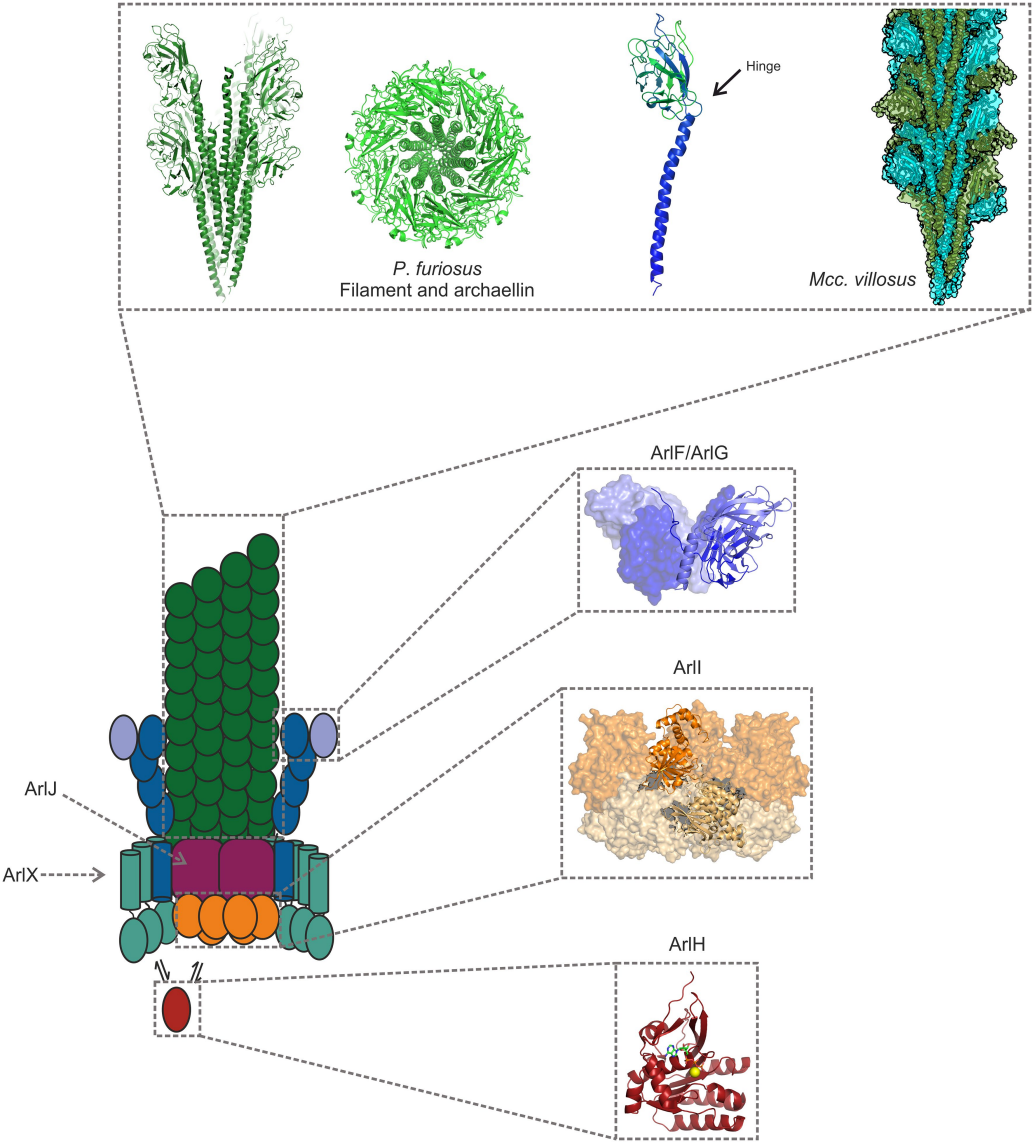
Model of the archaellum machinery. The filament consists of tadpole shaped archaellins (left), with an N-terminal α-helical tail and a β-sheet rich, globular head domain. The hydrophobic tails make up the core of the archaellum, while the hydrophilic heads face the filament’s exterior (Poweleit et al., 2016; Daum et al., 2017; Meshcheryakov et al., 2019; Gambelli et al., 2021). Two filaments are shown in the figure: one from *Pyrococcus furiosus*, and another from *Methanocaldococcus villosus* (PDB-7OFQ), a heteropolymeric filament. ArlG forms a filament that spans the pseudoperiplasm and that is tightly anchored to the S-layer by a heterotetramer consisting of ArlG and ArlF (Banerjee et al., 2015; Tsai et al., 2020; Umrekar et al., 2021). ArlI is the hexameric ATPase, which localises to the cytosolic side of the plasma membrane, probably owing to its interaction with the platform protein ArlJ, whose structure has not yet been determined (Reindl et al., 2013). ArlH is an auto-kinase that interacts with ArlI, modulating its activity and perhaps controlling when the ATPase assembles or rotates the filament (Chaudhury et al., 2016; Sousa Machado et al., 2021). The filament structure has been obtained from *P. furiosus* (5O4U; Daum et al., 2017), and the remaining structures from *Sulfolobus acidocaldarius* proteins 5TUG (Tsai et al., 2020); 4IHQ (Reindl et al., 2013); and 4YDS (Chaudhury et al., 2016).

The archaellum filament consists of helically organised proteins called archaellins. Archaellins are tadpole shaped proteins with a hydrophobic N-terminal tail and a hydrophilic, β-sheet-rich globular C-terminus (Figure 2). The hydrophobic N-termini of the archaellins form the core of the filament, while the (often N-glycosylated) C-termini face outwards (Poweleit et al., 2016; Daum et al., 2017; Meshcheryakov et al., 2019; Gambelli et al., 2021). The N- and C-termini are coupled by a flexible hinge region, which allows each archaellum to change its shape in bending and twisting archaella. In addition, the hydrophobic tail domains are able to slide and rotate past each other, adding to the flexibility of the filament (Gambelli et al., 2021).

In many species, the *arl* operon encodes multiple archaellins, which are likely the result of gene duplication events. These duplicates are usually not redundant, even in cases where their deletion does not result in defective archaellum biogenesis. For instance, in the halophile *Haloarcula marismortui* the two archaellins ArlA and ArlB are ecoparalogs, each forming a filament better adapted to different salinity conditions (Syutkin et al., 2014, 2019). In some other organisms, all archaellin homologues are necessary for the synthesis of functional archaella. This suggests that—at least in some archaea—different archaellins make up different parts of the archaellum.

Take the example of *H. salinarum*: this organism encodes five archaellin genes belonging either to the *arlA* (*arlA1* and *arlA2*) or *arlB* (*arlB1-3*) families (Gerl and Sumper, 1988; Gerl et al., 1989). Deletion of the *arlA* and *arlB* genes results in cells without archaella (Tarasov et al., 2000). In cells where only the *arlA* or the *arlB* gene is deleted, archaella still form. However, these cells show significantly hampered motility, indicating that both archaellins are important for proper archaellar function (Tarasov et al., 2000). Even within the *arlA* family there seems to be no redundancy: when only *arlA2* was disrupted in *H. salinarum*, the cells expressed only straight (rather than curved) filaments, resulting in decreased motility (Tarasov et al., 2000). Deleting *arlA1* and *arlA2* in *H. salinarum* resulted in a mutant that expressed only the archaellins *arlB1-3*. These cells formed short, curved filaments, corroborating the notion that these archaellins may form the cell-proximal region of the archaellum filament (Tarasov et al., 2000). Methanococcales also seem to assemble archaella in which some of the archaellins are minor components of the filament, forming hook-like, specialised structures in the cell-proximal region (Kalmokoff et al., 1988; Bardy et al., 2002).

In *Methanococcus voltae*, the minor archaellin ArlA is distributed along the filament (Bardy et al., 2002). Although disrupting *arlA* does not abolish archaella synthesis, it does decrease motility in this organism, suggesting an unknown, important function for this minor archaellin (Jarrell et al., 1996). *Pyrococcus furiosus*, which possesses the genes *arlB0*, *arlB1*, and *arlB2*, has an archaellum filament which appears to consist mostly of ArlB0, with ArlB1 and ArlB2 possibly localising to the ends of the filament (Näther-Schindler et al., 2014; Daum et al., 2017). Most recently, it has been shown that the archaellum filament of *Methanocaldococcus villosus* consists of two alternating subunits—ArlB1 and ArlB2 (Gambelli et al., 2021). A third archaellin (AlrB3) is encoded in the genome and it is not found in the main part of the filament, again suggesting a role in forming a terminal structure.

## The Archaellum Motor Complex

The rotation of the archaellum is driven by a motor complex that is embedded in the archaeal cell envelope. Analogous to the bacterial flagellum, the archaellar motor complex must have two components: a rotor and a stator. Torque is generated at the interface of a moving rotor and a stator that forms the bearing of the motor. In the flagellum, the stators MotA/MotB (or, in some species, PomA and PomB) also function as ion channels that convert an ion-motive force across the membrane into a conformational change, which in turn drives the rotation of the motor rings (Hu et al., 2021). As there is currently not a clear understanding of how the different protein components of the archaellum cooperate to generate torque, the identity and function of its motor and stator components is not fully understood. Here, we assess the structural, biochemical, and biophysical knowledge of the archaellum in order to present an informed guess at possible rotation mechanisms of the archaellum motor complex. A summary of structures solved for components of the archaellum machinery is shown in Figure 2.

### The Motor ATPase ArlI

ArlI is the only *bona fide* ATPase encoded in the *arl* operon. The enzymatic activity of ArlI from the archaeal species *Saccharolobus solfataricus* (formerly *Sulfolobus solfataricus*; Sakai and Kurosawa, 2018), *Sulfolobus acidocaldarius*, and *P. furiosus* has been characterised *in vitro* (Albers and Driessen, 2005; Ghosh et al., 2011; Chaudhury et al., 2016, 2018). As the only protein capable of hydrolysing ATP, ArlI is the sole candidate for powering the assembly and bidirectional rotation of the archaellum filament. The dual role of ArlI has been reinforced by the finding that the first 29 residues of *Sa*ArlI are essential for filament rotation but not for its assembly (Reindl et al., 2013). Despite being a soluble protein, ArlI is membrane-associated, and indeed tetraether lipids increase the ATPase activity of this protein (Albers and Driessen, 2005; Ghosh et al., 2011; Reindl et al., 2013). A structure with 2 Å resolution of *S. acidocaldarius* ArlI has resulted in the identification of an N-terminal three-helix bundle that is essential for the localisation of ArlI to the cell membrane (Reindl et al., 2013).

Monomeric ArlI has two distinct domains. These two domains are separated by a hinge, which confers some flexibility between them (Reindl et al., 2013; Mancl et al., 2016). Homology between ArlI and ATPases involved in T4P assembly and retraction hinted that ArlI might form hexameric oligomers (Berry and Pelicic, 2015), as later confirmed *in vitro* (Ghosh et al., 2011; Reindl et al., 2013; Chaudhury et al., 2018; Figure 2). Hexamerisation was found to be ATP-dependent, but ArlI from *P. furiosus* forms hexamers even without prior addition of this nucleotide (Mancl et al., 2016; Chaudhury et al., 2018). The X-ray structure of *S. acidocaldarius* ArlI shows a hexamer with 2-fold symmetry (Reindl et al., 2013), in which two protomers adopt an open, and the remaining four a closed, conformation. This is in line with biochemical evidence from ArlI from *P. furiosus* (Chaudhury et al., 2018): when MANT-ATP (a fluorescent analogue of ATP) is titrated in a reaction mixture containing ArlI, saturation is achieved when the concentration of MANT-ATP equals one third that of ArlI, indicating that only two ATP-binding pockets are available (Chaudhury et al., 2018).

The structure of ArlI from *S. acidocaldarius* has allowed the modelling of the conformational changes that occur in the hexamer upon ATP binding, hydrolysis, and product release. These molecular motions include a rotation of inter-subunit blocks, which comprise the N-terminus of one protomer in close interaction with the C-terminus of a neighboring protomer. This motion likely drives the insertion of archaellin subunits during filament biosynthesis and facilitates filament rotation (Reindl et al., 2013). These conformational changes are possibly relayed across the cell membrane by the protein ArlJ. ArlI needs to be switchable so that it can promote either archaellin insertion during filament growth or rotation of a mature filament. ArlH is the protein that may provide this switch.

### The Platform Protein ArlJ

The *arlJ* gene encodes a polytopic membrane protein homologous to the bacterial genes *pilC* and *gspF* belonging to the T4P and T2SS, respectively (Peabody et al., 2003). There is little experimental information about this protein except for it being essential for archaella assembly (Thomas et al., 2002; Chaban et al., 2007; Lassak et al., 2012). Bioinformatics predicts that ArlJ contains 7–9 transmembrane domains and conserved cytosolic loops (Thomas et al., 2001a; Ghosh and Albers, 2011). These cytosolic loops are rich in positively charged amino acids, which may interact with the negatively charged N-termini of ArlI (Ghosh and Albers, 2011). In *S. acidocaldarius*, ArlJ is unstable in the absence of ArlX, indicating that the two proteins interact (Lassak et al., 2012). ArlJ is presumably a platform protein during assembly and acts as a rotor that provides torque to the assembled archaellum filament (Jarrell et al., 2021). Notably, the homologous PilC protein has been suggested to rotate during extension and retraction of Type 4 pili (Chang et al., 2016), a mechanism which could conceivably have been adapted for the rotation of a filament during evolution. Moreover, it is possible that ArlJ interacts with the transmembrane domain of the putative stator subunit ArlG, which would be essential for ArlJ to act as a rotary component of the archaellum machinery.

### The Putative Regulator Protein ArlH

ArlH is an ATP-binding protein that is essential for archaella biogenesis (Thomas et al., 2001b; Lassak et al., 2012; Chaudhury et al., 2016; Li et al., 2020), but its mode of action remains largely unknown. Two experimentally determined ArlH structures are currently available, one from the crenarchaeon *S. acidocaldarius* and another from the euryarchaeon *Methanocaldococcus jannaschii* (Chaudhury et al., 2016; Meshcheryakov and Wolf, 2016). The two structures are similar. Both show a RecA fold consisting of a β-sheet sandwiched between α-helices. ArlH belongs to the KaiC-like ATPases, a group of proteins widespread in Archaea. Archaeal KaiC-like ATPases are homologous with the well characterised cyanobacterial KaiC, which has a central role in the regulation of the circadian rhythm in these organisms (Johnson et al., 2017; Makarova et al., 2017). ArlH binds ATP, which is required for the biogenesis of the archaellum filament, presumably because only ATP-bound ArlH is able to stimulate the ATPase activity of ArlI (Chaudhury et al., 2016, 2018).

ArlH itself does not hydrolyse ATP (Chaudhury et al., 2016, 2018; Meshcheryakov and Wolf, 2016). Instead, ArlH exhibits auto-phosphorylation activity (Sousa Machado et al., 2021). ArlH has been shown to interact with other archaellum motor components, including ArlI (Chaudhury et al., 2016, 2018; Sousa Machado et al., 2021), ArlX (Banerjee et al., 2013; Chaudhury et al., 2016), and ArlCDE (Li et al., 2020). Phosphorylation of ArlH seems to influence both its oligomerisation and how it interacts with ArlI: the interaction between ArlI and ArlH is strongest when ArlH is not phosphorylated, and under these circumstances, ArlH adopts a hexameric form (Sousa Machado et al., 2021). When ArlH autophosphorylates, the hexameric oligomer disassembles from the ArlI/ArlH complex (Sousa Machado et al., 2021). It has been hypothesised that this is the signal that switches the archaellum machinery from filament assembly to filament rotation. As ArlH has been shown to interact with ArlX/ ArlCDE, it may remain bound to these proteins after phosphorylation and dissociation from ArlI (Banerjee et al., 2013; Li et al., 2020).

### The Putative Stators ArlFG

ArlF and ArlG are both periplasmic components of the archaellum motor complex. On the level of structure and sequence, both proteins show key similarities with archaellins, despite lacking the signal peptide that is characteristic for the latter (Tsai et al., 2020). Biochemical data indicate that ArlF interacts with S-layer proteins, which suggests a role for ArlF in anchoring the motor complex to the cell surface (Banerjee et al., 2015). Later, it was found that ArlG is secreted to the periplasm after being processed, but it was also seen that the processing of ArlG is not dependent on PibD/ArlK (Tsai et al., 2020). ArlG forms filaments, which can be capped by a heterotetramer formed by two ArlG and two ArlF protomers. These observations led to a model in which an ArlG filament spans the pseudoperiplasm, at which point an ArlF/ArlG heterotetramer connects the filament with the S-layer (Tsai et al., 2020; Umrekar et al., 2021). The role of these proteins is likely 2-fold; the ArlF/ArlG complex provides a scaffold around the motor complex, allowing for the rotation of the archaellar filament without cellular disruption. In addition, ArlF and ArlG act as a stator against which the motor rotates (Umrekar et al., 2021). The S-layer does not seem to be essential for the assembly of the archaellum filament, as *Sulfolobus islandicus* cells lacking an S-layer still synthesise archaella. However, these archaella are unable to rotate (Tsai et al., 2020).

### The Cytosolic Ring ArlX

The *arlX* gene encodes a predicted membrane protein with a single α-helical transmembrane domain. ArlX is essential for archaella biogenesis (Banerjee et al., 2012; Lassak et al., 2012). The cytosolic domain of *S. acidocaldarius* ArlX (*Sa*ArlXc) has been purified and shown to form an oligomeric ring structure with variable diameter, averaging 30 nm (Banerjee et al., 2012). Moreover, ArlXc was shown to interact with the cytosolic components of the *S. acidocaldarius* archaellum motor complex, ArlI and ArlH, with the latter localising inside the ring formed by ArlX (Banerjee et al., 2012; Chaudhury et al., 2016). Genetic data suggest that ArlX is stabilised by archaellins and ArlJ (Lassak et al., 2012). In combination, these data suggest that ArlX forms a stabilising ring around a central complex consisting of ArlJ, ArlI, and ArlH (Banerjee et al., 2012). In the same study, it was also suggested that ArlX may have a stator-like role in the archaellum motor complex (Banerjee et al., 2012).

### ArlCDE and the Polar Cap

ArlC/D/E are thought to replace ArlX in the motor complexes of non-crenarchaeal species. Despite its possibly analogous role to ArlX, ArlC, D, and E lack the transmembrane domain found in ArlX. ArlC, D, and E are frequently found as fusion (e.g., ArlDE or ArlCDE), indicating that they are functionally interdependent and physically interact, which has been experimentally demonstrated for ArlCE and ArlD of *Haloferax volcanii* (Ng et al., 2006; Makarova et al., 2016; Li et al., 2020).

ArlCDE have been shown to interact directly with CheF, presumably because ArlCDE act as a switch complex that regulates motor activity upon chemoreceptor activation (Schlesner et al., 2009; Li et al., 2020). The interaction of ArlCDE with the core motor complex seems to be mediated by ArlH, as recently shown in *H. volcanii* (Li et al., 2020). CryoET of the motor complex from *P. furiosus* has led to the suggestion that ArlCDE (in this organism, ArlC and ArlDE) form a cytosolic ring around ArlI and ArlH. Furthermore, ArlC and ArlDE might interact with a cytosolic structure called the polar cap (Briegel et al., 2017; Daum et al., 2017). This polar cap is a cone-shaped, sheet-like and most likely proteinaceous structure that co-localises with the archaellated cell pole. The polar cap appears to be a hallmark of Euryarchaeota and was observed in early electron microscopy studies (Koval and Jarrell, 1987; Gongadze et al., 1993; Kupper et al., 1994). Until recently, the polar cap was assumed to correspond to chemosensory arrays (Briegel et al., 2015). Detailed analysis of the archaellum motor complex in *P. furiosus* and *Thermococcus kodakarensis* by cryogenic electron tomography (CryoET; Briegel et al., 2017; Daum et al., 2017) revealed that the polar cap is closely associated with the archaellum motor, suggesting that both may be physically and functionally linked.

Various functions have been suggested for the polar cap. For example, the polar cap may act as an organising centre that ensures the polar organisation of the archaellar bundle. In addition, it has been proposed that the polar cap may serve as a cytoplasmic anchor for archaella motor complexes in the absence of a membrane anchor in the putative ArlCDE stator (Briegel et al., 2017; Daum et al., 2017; Jarrell et al., 2021). Lastly, as chemosensory arrays have been observed to associate with the polar cap, it may be that the polar cap forms a relay between chemoreceptors and the archaellar motors.

### Models Derived From CryoET

Despite various pieces of evidence about how the subunits of the archaellum motor interact with each other, the structure of the assembled machinery remains largely elusive. In 2017, a first three-dimensional *in situ* map of the motor complex in context with the archaellum filament, the S-layer, and the polar cap was published (Daum et al., 2017). The map showed the motor as a bell-shaped complex that projects from the cell membrane into the cytoplasm. This central complex is connected to a surrounding cytosolic ring, which itself does not appear to be connected to the cell membrane. By fitting the atomic model of ArlI from *S. acidocaldarius* and a 6-fold symmetric model of ArlH into the map, a structure for an ArlI-ArlH double-ring complex was suggested (Daum et al., 2017). In this model, ArlI attaches to the membrane *via* its N-terminal protrusions, while ArlH is associated with the opposite surface of the molecule.

Because of its position, its connection with the ArlI-ArlH complex, and its similarity with cryoEM maps of ArlX, it was suggested that the cytosolic ring may correspond to ArlC,D/E (Daum et al., 2017) This ring was not seen in tomograms of *T. kodakarensis* (Briegel et al., 2017), which could either be a consequence of species-specific variation of the archaellum motor architecture as seen in bacteria (Rossmann and Beeby, 2018), flexibility of the ring complex, or differences in the sample preparation method.

The motor complex is juxtaposed to the polar cap, suggesting a physical connection between them. However, because of limitations in resolution, no connections between polar cap and archaellum motor were resolved. Similarly, the membrane-embedded protein ArlJ and the potential ArlF/G stator subunits were not discernible.

## The Biophysics of the Archaellum Motor

Various biophysical parameters of archaellum filament rotation have been calculated for the motor of the halophiles *H. salinarum* and *H. volcanii* (Kinosita et al., 2016, 2020; Iwata et al., 2019). Both these organisms harbour the putative switch complex ArlCDE, a chemotaxis system and, at least for *H. salinarum*, a polar cap (Kupper et al., 1994). In the first detailed biophysical analysis of the rotating archaellum, the motor appeared to be stepping at 60°–36° intervals (6–10 steps per revolution; Kinosita et al., 2016). The number of steps during the revolution of a molecular motor can be correlated with the rotary mechanism. As described below, the ATPase that powers the archaellum forms a hexamer (Ghosh et al., 2011; Reindl et al., 2013; Chaudhury et al., 2018). The discrete 60° steps could thus correspond to the hydrolysis of one ATP molecule per monomer, with the hydrolysis of six ATP molecules per revolution. The ~36° steps, on the other hand, were hypothesised to be either associated with the hypothetical presence of 9–10 monomers of the modulator ArlH in the motor complex (as observed by Chaudhury et al., 2016), or motor slippage. Recent evidence suggests that ArlH forms a hexamer in the motor (Sousa Machado et al., 2021), indicating the 9–10 stoichiometry of ArlH observed in previous single particle cryoEM data (Chaudhury et al., 2016) may have been an artefact of sample preparation. A more recent calculation indicated that the torque output of *H. salinarum* archaella is 160 pN.nm regardless of its load (Iwata et al., 2019). This torque value allowed the energy per revolution to be determined at 1,000 pJ, which is approximately twice the energy released by the hydrolysis of six molecules of ATP. This study suggested that more than six molecules of ATP are hydrolysed per revolution, and a model to account for this observation was proposed. According to this model, the membrane protein ArlJ is assumed to have *n*-fold symmetry, in which specific portions of ArlJ are in contact with *n* active sites of the ATPase ArlI. Each rotation step would therefore result in the hydrolysis of *n* ATP molecules simultaneously. By considering a motor composed of ArlJ_2_:ArlI_6_—which is likely, based on research on the platform protein of other TFF (Karuppiah et al., 2010; Bischof et al., 2016; Chang et al., 2016; Van Putte et al., 2018)—12 molecules of ATP would be hydrolysed per revolution. The energy released from the hydrolysis of these molecules would then result in a motor efficiency of ~100%. Other symmetries of ArlJ are also possible, with higher *n* resulting in lower motor efficiency.

Experiments on the archaellum of *H. volcanii* resulted in similar torque and energy values (Kinosita et al., 2020). The *in situ* enzymatic activity of ArlI from *P. furiosus* was found to be too low to allow for the required turnover of ATP to rotate the filament—a surprising finding given that the activity of ArlI from *P. furiosus* is reported to be 250 times higher than that measured for the ArlI of *S. acidocaldarius* (Ghosh et al., 2011; Chaudhury et al., 2018; Kinosita et al., 2020). The function of ArlI could be stabilised and stimulated *in vivo* in the presence of other motor components, as suggested by Kinosita et al. (2020). The calculations determining the number of ATP molecules hydrolysed per revolution according to the estimated torque assume a certain value of free energy per ATP molecule (Kinosita et al., 2020). Since free energy depends on temperature, it is also possible that calculations based on standard free energy of hydrolysis do not translate appropriately for hyperthermophilic organisms.

## AlphaFold Predictions of the Motor Core Complex

The core of the archaellum motor is formed by the ATPase ArlI and the platform protein ArlJ, an architecture likely similar to that of other TFFs (Denise et al., 2020).

**Figure 3:**
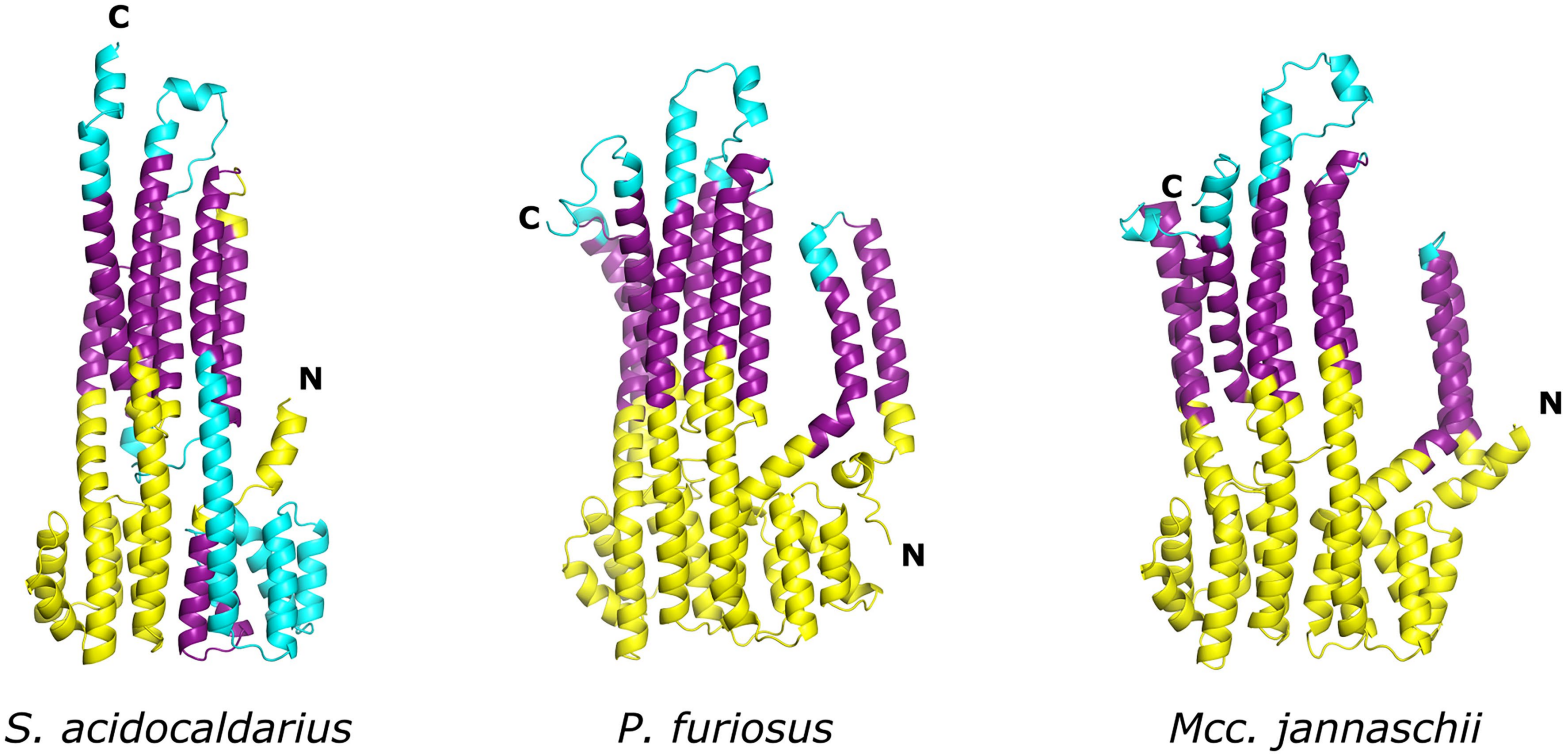
Structure of ArlJ homologues predicted with AlphaFold. AlphaFold (Jumper et al., 2021) predictions of ArlJ from homologs of *Sulfolobus acidocaldarius*, *Pyrococcus furiosus*, and *Methanocaldococcus jannaschii*. Transmembrane regions were predicted with TMHMM. The cytosolic, transmembrane, and extracellular regions are, respectively, coloured yellow, purple, and cyan.

**Figure 4:**
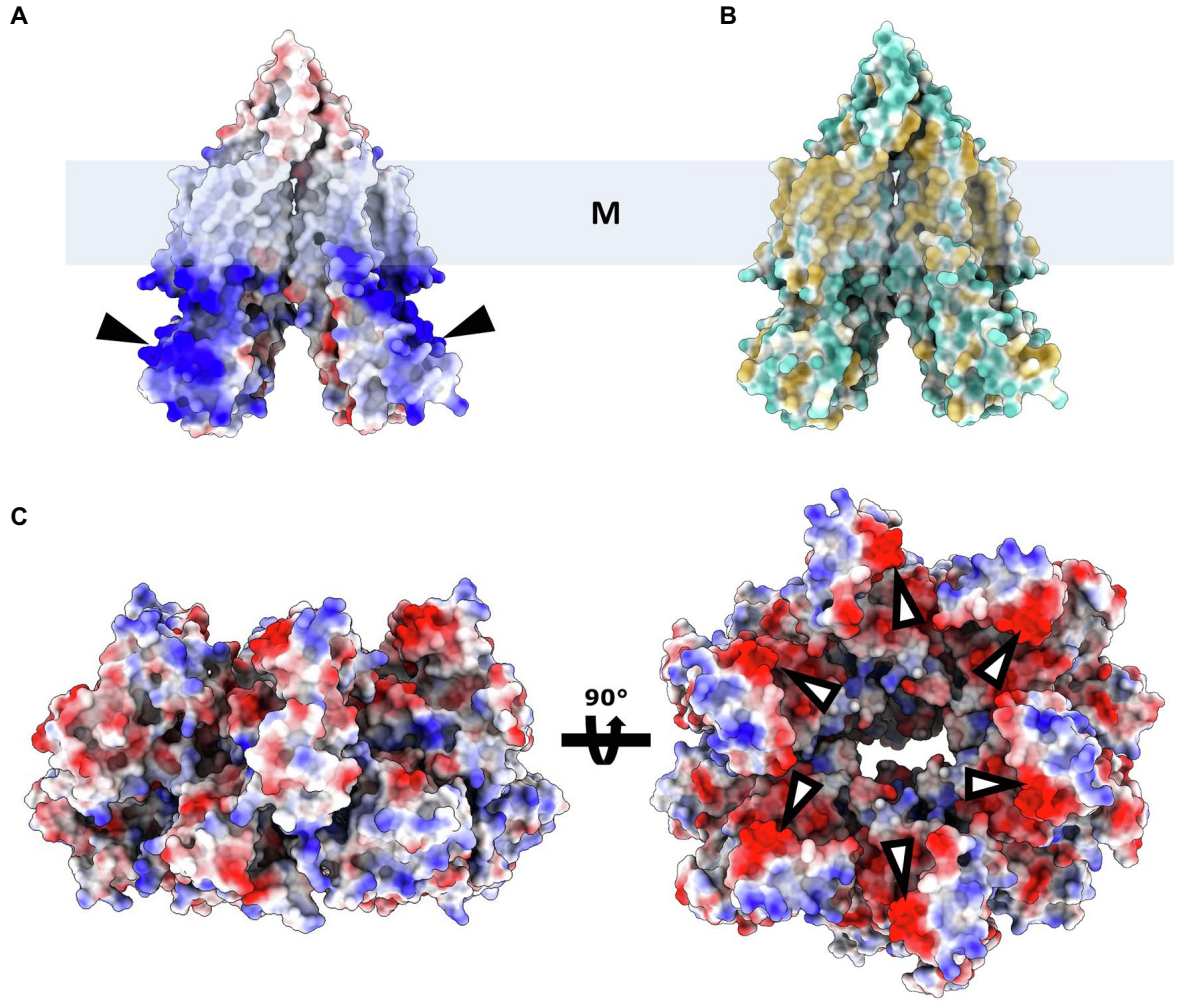
ArlJ modelled as a dimer. **(A)**, surface representation of electrostatic potential (red, negative; white, neutral; and blue, positive local charges). **(B)**, surface representation of ArlJ coloured by hydrophobicity (blue, hydrophilic; yellow, hydrophobic). The position of the membrane (represented by the box M) can be inferred from the location of the hydrophobic belt **(B)**. According to the positive-inside rule, the positively charged domains **(A)** are likely located in the cytoplasm. **(C)**, ArlI (PDB-4IHQ) surface representation coloured by electrostatic potential. The complementary local charges in ArlJ and ArlI (black and white arrows, respectively) hint at electrostatic interactions between both proteins.

**Figure 5:**
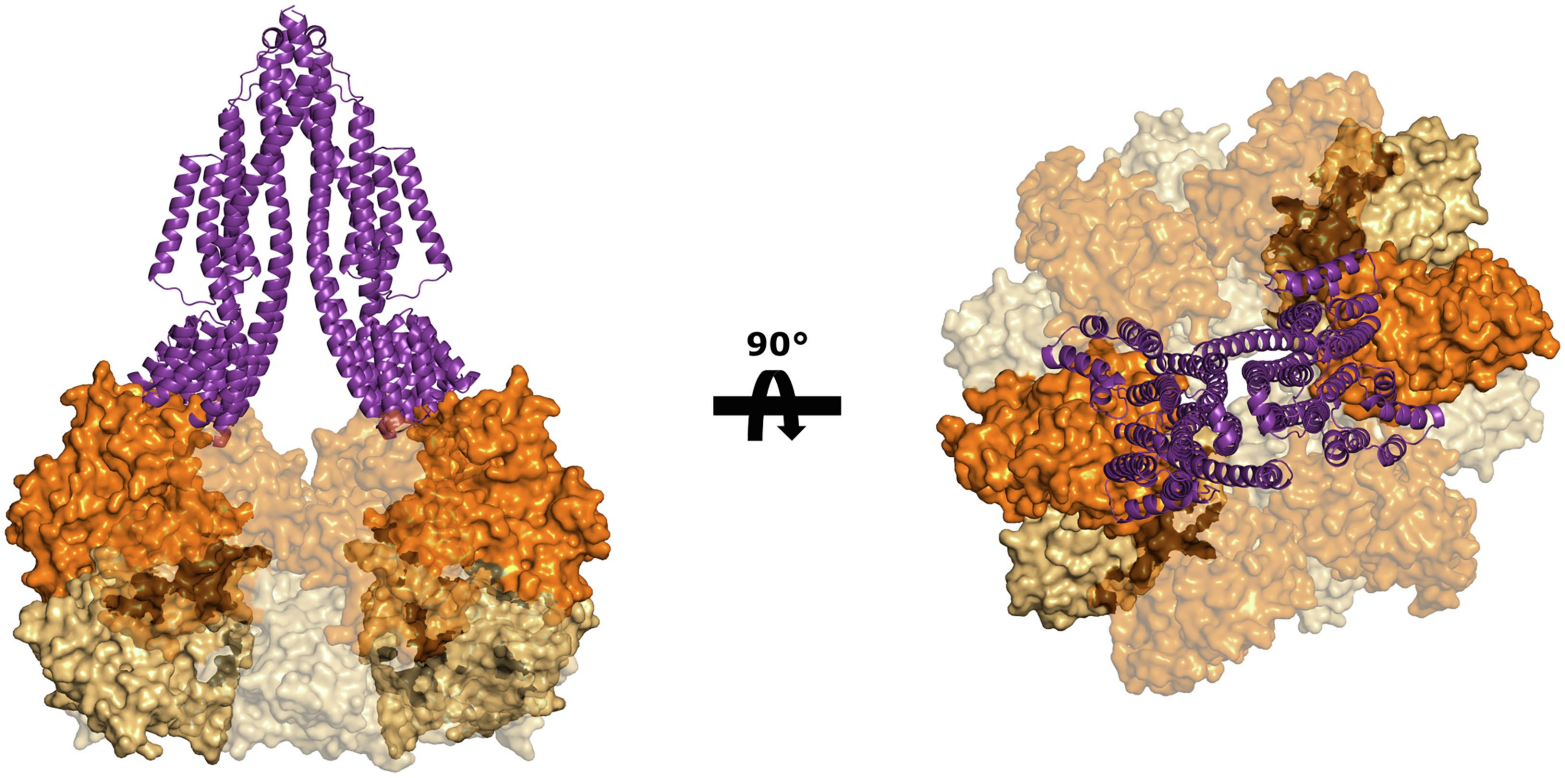
Putative interaction between ArlJ and ArlI. The charge complementarity between the N-terminus of ArlI (PDB-4IHQ) and the cytosolic domain of the AlphaFold prediction of the ArlJ dimer from *S. acidocaldarius* suggests how the two proteins may interact. The model is shown in side view (left) and as seen from the pseudo-periplasm (right). In the model (highlighted), which would have important consequences for the mechanism of rotation.

Despite the lack of biochemical and structural data on ArlJ—which was also not observed in the CryoETs—it is currently possible to predict the overall organisation of this core with the aid of AlphaFold-predicted structures of ArlJ (Jumper et al., 2021). The structure of ArlJ was predicted for *S. acidocaldarius* and for *P. furiosus*, and the resulting structures were compared with the AlphaFold-predicted ArlJ structure of *Mcc. jannaschii* available in UniProt. Overall, the three ArlJ homologs share a similar predicted structure (Figure 3). However, compared to ArlJ of *S. acidocaldarius*, the euryarchaeotic homologs (*P. furiosus* and *Mcc. jannaschii*) show an N-terminal extension. Alignment of ArlJ primary sequences has previously resulted in the identification of conserved cytosolic loops (Ghosh and Albers, 2011). These loops are rich in often conserved lysines and arginines, which are positively charged at the approximately neutral cytoplasmic pH. Based on the positive-inside rule for the topology of membrane proteins (von Heijne, 1992) and the putative interaction surface mediating ArlJ and ArlI interaction (Ghosh and Albers, 2011; Reindl et al., 2013), it is likely that this region faces the cytoplasm and interfaces with ArlI. These observations are in accordance with the TMHMM-predicted cytosolic, transmembrane, and extracellular regions, which are differentially coloured in the models in Figure 3 (Sonnhammer et al., 1998; Krogh et al., 2001). Alphafold 2 was used to predict ArlJ from *S. acidocaldarius* and *P. furiosus* as a dimer, resulting in a heart-shaped structure (Figure 4). Calculating the hydrophobicity and charge distribution suggest the position of the membrane (Figure 4), as well as a putative electrostatic interface between ArlJ and ArlI (Figure 5). The relative orientation of ArlJ and ArlI suggests how a dimeric ArlJ may interact simultaneously with two monomers of ArlI. This has potential implications for the mechanism of archaellum rotation, as we explore below. The predicted structure shows a cleft between the two ArlJ monomers. It is conceivable that this cleft could serve as a lateral gate for incoming archaellins. Clamshell-like conformational changes within the ArlJ dimer could then facilitate the transfer of archaellins into the growing filament. However, it remains elusive how the mature archaellum filament remains anchored by the ArlJ dimer post assembly.

## Models for a Rotating Archaellum

Much information has been gathered about the individual components of the archaellum over the past years (see Jarrell et al., 2021, for a comprehensive review), but due to limited resolution of the available structures (Briegel et al., 2017; Daum et al., 2017), as well as incomplete biochemical understanding of the machinery, it is not yet possible to build a convincing and comprehensive mechanistic model to explain how the archaellum rotates. In particular, the positions and stoichiometry of some if its component proteins, such as ArlJ, ArlF, AlrG, and ArlCDE are currently unknown.

The first issue to tackle while devising possible mechanisms for archaella rotation is establishing, which archaellar components remain static and which ones move. According to the current models (Jarrell et al., 2021), ArlF and ArlG can be safely assumed to act as stators because the ArlG filament is capped by an ArlGF tetramer that is tightly bound to the S-layer (Banerjee et al., 2015; Tsai et al., 2020). Conversely, ArlJ is predicted to localise at the interface between the ATPase ArlI and the filament; therefore, ArlJ must be able to rotate in order to convey torque to the filament. Beyond this, it is more difficult to predict whether the remaining subunits of the machinery rotate or not. Thus, we propose two possible hypotheses that start from the assumption that ArlJ is a rotor and ArlFG are stators.

**Figure 6:**
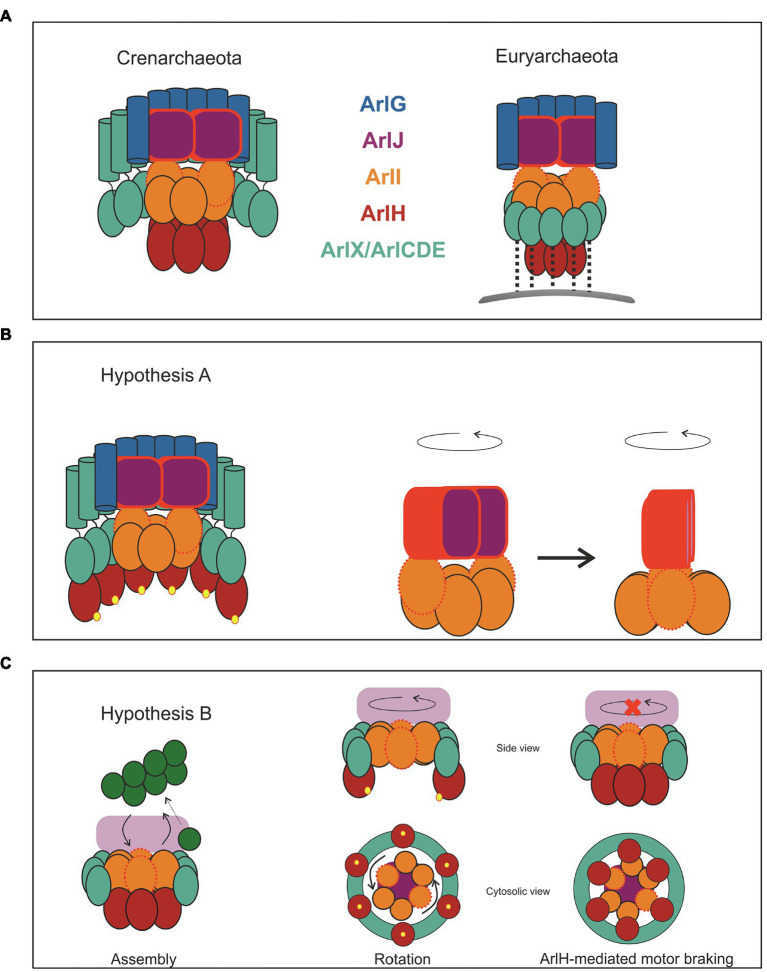
Two hypotheses for archaellum rotation. Despite the differences between the crenarchaeal and euryarchaeal motor, we suggest two hypotheses for the rotary motion of this motor, which are applicable to both phyla. **(A)** Two schemes representing the crenarchaeal and the euryarchaeal motors. The motor is primed for archaella synthesis when ArlH is interacting with ArlI. Once ArlH is phosphorylated (represented by yellow spheres), this protein is either ejected from the motor complex or remains bound to ArlX or ArlCDE. In **(B,C)**, only the crenarchaeal motor is shown for simplicity. In hypothesis A, only ArlJ rotates. ArlJ, likely as a dimer, has high affinity for ArlI subunits in open conformation (red, dotted outline) and binds only weakly to ArlI subunits in the closed conformation. ATP hydrolysis causes ArlI subunits to switch from the closed to the open conformation, so sequential ATP hydrolysis causes subunits of closed and open conformation to cyclically go through the otherwise static hexamer. The binding preference of ArlJ to the open conformation of ArlI thus causes ArlJ to rotate by 60 degrees for each ATP hydrolysis step. The rotation of ArlJ thus causes the gyration of the filament. In **(B)**, both ArlI and ArlJ rotate. The side and bottom views of the motor are shown for clarity. The sequential ATP hydrolysis-induced conformational changes within ArlI push against the ArlX or ArlCDE stators, relax, and push again. The repetition of this process results in ArlI rotation. ArlI tightly interacts with ArlJ, causing it to rotate, which in turn transmits torque to the filament. Through the interaction of ArlH with ArlI **(A,B)** the conformational changes in ArlI result in piston-like motions in ArlJ, which push archaellins from the membrane into the growing filament. In **(B)** we show how ArlH may act as a motor brake: when ArlH is dephosphorylated (presumably by an unknown factor), this protein interacts once more with ArlI. Since ArlI, ArlH, and ArlX/CDE form now a triple complex, ArlI is unable to rotate against the ArlX/CDE surface.

In the first hypothesis (Hypothesis A, Figure 6), we consider that all proteins remain static with the exception of ArlJ. The N-terminus of ArlI interacts with the cytosolic loops of ArlJ. Assuming that ArlI is a hexamer and ArlJ is a dimer, two opposite monomers of ArlI interact each with an ArlJ monomer, as suggested in the model in Figure 5. This interaction is favoured when the two FlaI monomers are in the open state. Upon ATP hydrolysis, a conformational change occurs (Reindl et al., 2013), which transfers the ArlJ loops to the subsequent ArlI monomers that will change to the open state. Upon repeated ATP binding and hydrolysis, these conformational changes cycle through the otherwise static ArlI hexamer. The sequential binding and resulting transfer of ArlJ to the two open ArlI monomers therefore results in rotation of the ArlJ dimer, which in turn drives the rotation of the filament (Figure 6).

In the second hypothesis (Hypothesis B, Figure 6), both ArlJ and ArlI rotate. In this scenario, ArlI and ArlJ bind tightly, independent of the catalytic state of ArlI. ArlI is surrounded by either ArlX (in Crenarchaeota) or ArlCDE (in Euryarchaeota), and it interacts loosely with this ring. The conformational changes induced by ATP-hydrolysis result in the C-termini of opposing ArlI monomers pushing outwards and against the ArlX/ArlCDE ring, generating torque that results in rotational movement of ArlI, and consequently of ArlJ and the attached filament (Figure 6). For both hypotheses, the torque is assumed to be generated by the sequential rotary mechanism of ATP hydrolysis in the ArlI ring, as proposed for PilB/PilF, the ATPase that drives the assembly of T4P in *Thermus thermophilus* (Mancl et al., 2016).

Although the function of ArlH remains largely elusive, it has been proposed that this protein is involved in switching the archaellum machinery between filament assembly and rotation. ArlH is essential for archaella assembly (Thomas et al., 2001b; Lassak et al., 2012), but there are no data regarding its relevance for the rotation of the filament, suggesting that ArlH is only essential for archaella assembly. This possibility is supported by the observation that upon autophosphorylation, ArlH ceases to interact with ArlI (Sousa Machado et al., 2021).

According to Hypothesis A (Figure 6), ArlH may determine how ArlI interacts with ArlJ. In its unphosphorylated state, ArlH would promote an ArlI-ArlJ complex that catalyses filament assembly. Upon autophosphorylation, ArlH would be ejected from the ArlI-ArlJ complex (or perhaps remain attached to ArlX/ArlCDE). Dislocation of ArlH would in turn switch the ArlI-ArlJ complex from assembly to rotation mode. The corollary of this hypothesis is that filament assembly would last for as long as it takes for ArlH to autophosphorylate and that the kinetics of this process determines the length of the filament. Assuming that during archaellum assembly the filament does not rotate, ArlH may also function as a brake. ArlH remains bound to ArlCDE in close proximity to the motor, as has been reported for *H. volcanii* (Li et al., 2020); if ArlH can be dephosphorylated, it would be able to re-associate with ArlI, stopping the motor and possibly resulting in a switch in the direction of rotation to regulate forwards and backwards swimming motion.

Hypothesis B also suggests a mechanism for archaellum assembly. Here, un-phosphorylated ArlH would connect ArlI firmly to ArlCDE/ArlX, preventing the rotation of the ATPase. In this configuration, the conformational changes within the ArlI hexamer may cause and up-and-down motion in ArlJ, scooping archaellin monomers from the membrane and inserting them into the growing filament.

## Conclusion

Recent years have seen increased research into the archaellum machinery, with a particular focus on solving the structures of its components (Reindl et al., 2013; Banerjee et al., 2015; Chaudhury et al., 2016; Meshcheryakov and Wolf, 2016; Poweleit et al., 2016; Briegel et al., 2017; Daum et al., 2017; Tsai et al., 2020; Gambelli et al., 2022). Despite all of these efforts, a high-resolution structure of the entire machinery has so far not been achieved, meaning that the molecular mechanism of the archaellum remains largely unknown. However, drawing on accumulating knowledge about its structural components and with the aid of novel structural prediction algorithms, it is possible to piece together a picture about the rotary mechanism of the archaellum. We hope that the working models that we propose in this review will guide and fuel future research that will ultimately lead to a full understanding of this fascinating molecular machine.

## Author Contributions

JS created the models for archaellum rotation and predicted the topology of the ArlJI complex. BD predicted the structures of ArlJ with AlphaFold-2. JS, S-VA, and BD discussed and contributed to the proposed models and wrote the manuscript. All authors contributed to the article and approved the submitted version.

## Funding

JS was supported by the Collaborative Research Centre SFB1381 funded by the Deutsche Forschungsgemeinschaft (DFG, German Research Foundation)—Project-ID 403222702—SFB1381. This study was supported in part by the Excellence Initiative of the German Research Foundation (GSC-4, Spemann Graduate School) and in part by the Ministry for Science, Research and Arts of the State of Baden-Wuerttemberg. BD was funded by the European Research Council (ERC) under the European Union’s Horizon 2020 Research and Innovation Programme (grant agreement No 803894).

## Conflict of Interest

The authors declare that the research was conducted in the absence of any commercial or financial relationships that could be construed as a potential conflict of interest.

## Publisher’s Note

All claims expressed in this article are solely those of the authors and do not necessarily represent those of their affiliated organizations, or those of the publisher, the editors and the reviewers. Any product that may be evaluated in this article, or claim that may be made by its manufacturer, is not guaranteed or endorsed by the publisher.
